# Axonal anisotropy and connectivity inhomogeneities in 2D networks

**DOI:** 10.1186/1471-2202-13-S1-P145

**Published:** 2012-07-16

**Authors:** Sarah Jarvs, Samora Okujeni, Steffen Kandler, Stefan Rotter, Ulrich Egert

**Affiliations:** 1 Bernstein Center Freiburg, University of Freiburg, Freiburg, 79104, Germany; 2 Faculty of Biology, University of Freiburg, Freiburg, 79104, Germany; 3 Department of Biomicrotechnology, IMTEK, University of Freiburg, Freiburg, 79096, Germany

## 

Cultured neuronal networks are an interesting experimental model in which neurons are freed from cortical architecture and plated on microelectrode arrays (MEA). Present in their dynamics are periods of strongly synchronized spiking by the network, termed 'bursting', whose role is not understood but dominates network dynamics and, due to its resistance to attempts to remove it [1], has been suggested to be an inherent feature in their dynamics. Bursts have been demonstrated to contains distinct spatiotemporal motifs, repudiating the possibility that they are random or chaotic activity. However, the speeds of these propagating wavefronts has been measured as 5-100mm/s [2], and hence much faster than can be accounted for by local connectivity [3].

In attempting to represent cultured networks using 2D network models, typical connectivity models, such as small-world, prove to be insufficient for recreating some of the distinct phenomena associated with the dynamics of cultured networks, noticeably the fast propagation speeds.

Here, we introduce a simple but biologically plausible connectivity model that is able to reproduce this phenomena. We extend it to incorporate some of the subtle structural inhomogeneities observed experimentally to investigate their implications for network dynamics. We demonstrate that these inhomogeneities strongly facilitate the propagation of activity as well as being responsible for emergence of distinct burst motifs. Importantly, our model confirms that bursts are indeed an inherent feature of such networks, as they are an inescapable by-product of network connectivity and structure.
